# Hearing From Men Living With HIV: Experiences With HIV Testing, Treatment, and Viral Load Suppression in Four High-Prevalence Countries in Sub-Saharan Africa

**DOI:** 10.3389/fpubh.2022.861431

**Published:** 2022-05-16

**Authors:** John Mark Wiginton, Sanyukta Mathur, Ann Gottert, Nanlesta Pilgrim, Julie Pulerwitz

**Affiliations:** ^1^Department of Health, Behavior and Society, Johns Hopkins University, Baltimore, MD, United States; ^2^Population Council, Washington, DC, United States

**Keywords:** men living with HIV, qualitative, sub-Saharan Africa (SSA), HIV care continuum, facilitators

## Abstract

Engaging men in HIV services remains a challenge across sub-Saharan Africa. There is a critical need to better understand facilitators of men's successful engagement with HIV services and assess if there are similarities across contexts. We conducted in-depth interviews and focus group discussions with 92 men living with HIV (MLHIV) across Malawi, Uganda, South Africa, and Eswatini, most of whom had been diagnosed with HIV within the last 5 years. We coded interviews for themes using a constant-comparative approach. We contextualized our findings within a socioecological framework. HIV testing was primarily motivated by illness (individual level), though illness was sometimes accompanied by prompting and support from healthcare providers and/or intimate partners. Once diagnosed, nearly all participants reported immediate linkage to care, initiation of antiretroviral therapy (ART), and subsequent ART adherence. ART initiation and adherence were facilitated by men's sense of agency and ownership over their health (individual level), social support from intimate partners, friends, and family (interpersonal/network level), supportive-directive counseling from healthcare providers (institutional/health systems level), and male-friendly services, i.e., rapid, respectful, private (institutional/health systems level). Health literacy regarding viral suppression (individual level), strengthened by patient-provider communication (institutional/health systems level), was highest in Uganda, where most men could discuss viral load testing experiences, report their viral load status (most reported suppressed), and demonstrate an understanding of treatment as prevention. Elsewhere, few participants understood what viral load suppression was and even fewer knew their viral load status. Our findings reveal socioecological-level facilitators of men's progress across the HIV-care continuum. Programs may want to leverage facilitators of ART initiation and adherence that span socioecological levels—e.g., healthcare ownership and agency, social support, supportive-directive counseling—and apply them to each end of the continuum to encourage early HIV testing/diagnosis and improve health literacy to help men understand and achieve viral load suppression.

## Introduction

Engaging men in HIV services in sub-Saharan Africa [SSA] continues to pose a challenge to HIV prevention and control efforts (1). Compared with women, men remain less likely to be engaged in services at each stage of the HIV-care continuum (2–5) and more likely to die of AIDS-related illnesses (6–8). Limited engagement of men, particularly in regions of SSA with the heaviest HIV burden, threatens achievement of the UNAIDS 90-90-90 targets locally and globally (1, 9). Furthermore, with recently implemented test-and-treat protocols and rapidly evolving ART regimens and healthcare system approaches, an up-to-date understanding of care and treatment experiences among men living with HIV (MLHIV) is needed (1, 9, 10).

Research has documented several factors that contribute to men's low uptake of HIV services in SSA, including community-level barriers, such as masculine norms and stigma (2, 7, 11–14), institutional/health systems-level barriers, such as privacy/confidentiality issues, long wait times, inflexible hours of operation, and negative interactions with providers (15–20), interpersonal-level barriers, such as social network/peer influences (21–23), and individual-level barriers, such as fear and denial of HIV and ART (1, 2, 24). These findings also demonstrate the disproportionate focus on what has not worked for men rather than on what has, fueling narratives that scapegoat men for lack of engagement in HIV care (25). By reconceptualizing men as a unique population with their own health needs and interests, health systems and providers can better respond and tailor services to meet men where they are (25).

Now in the fourth decade of the HIV epidemic, hearing from men about what facilitates their engagement with HIV services is critical to improving HIV programming for men. Over the last decade there has been a surge in efforts to reach men with HIV testing and treatment services, especially in Eastern and Southern Africa (1). We conducted a multi-country qualitative study with MLHIV in Uganda, Malawi, South Africa, and Eswatini—among the countries in SSA hardest hit by the epidemic—in order to contribute to the evidence base around how to strengthen HIV services for men across the HIV-care continuum. We applied a socioecological frame (26) to contextualize men's experiences at multiple levels—individual, interpersonal/network, community, and institutional/health system—across the HIV-care continuum (27–30). We focus on facilitators of men's entry into HIV testing and care to provide insights into what has worked to engage men as clients to improve their health.

## Materials and Methods

Data come from a portfolio of implementation science studies conducted with men in countries in Eastern Africa (Malawi, Uganda) and Southern Africa (Eswatini, South Africa) in 2018 and 2019 (31) (Table 1), following adoption of universal test-and-treat policies in these countries. These sites were part of the DREAMS (Determined, Resilient, Empowered, AIDS-free, Mentored, Safe) partnership program, which was supported by the United States President's Emergency Plan for AIDS Relief (PEPFAR) and focused on reducing HIV risk and incidence among adolescent girls, young women, and their male partners. The specific studies upon which the present paper is based sought to understand relationships between men and their sexual partners, particularly adolescent girls and young women disproportionately vulnerable to HIV (32, 33), as well as men's use of HIV testing, care, and treatment services. This paper focuses on the subsample of MLHIV interviewed in these studies.

**Table 1:** Selected methods from qualitative research with MLHIV in four sub-Saharan African countries, 2018–2019 (*N* = 92).

	**Malawi** **(*n =* 48)**	**Uganda** **(*n =* 17)**	**South Africa** **(*n =* 20)**	**Eswatini** **(*n =* 7)**
Study sites	Machinga district Zomba district	Gulu district Mukono district Sembabule district	Two informal settlements in eThekwini District (Durban) in KwaZulu-Natal Province	Multiple Inkhundla (districts)
Recruitment strategies	Community support groups for MLHIV, facilitated by DREAMS implementing partners & local clinics	Referrals from DREAMS implementing partners^a^ and men's female partners participating in DREAMS programming	Referrals from DREAMS implementing partners^a^; Self-reported positive HIV status on parent study survey^b^; HIV service sites	Referrals from DREAMS implementing partners^a^; Self-reported positive HIV status on parent study survey^b^
Data collection	In-depth interviews (*n =* 15) and four focus group discussions (*n =* 33)	In-depth interviews (*n =* 48)	In-depth interviews (*n =* 20)	In-depth interviews (*n =* 7)
Incentives^c^	USD5	USD5	USD3	USD5
Ethical approval	College of Medicine Research & Ethics Committee at University of Malawi	Makere University School of Public Health Institutional Review Board; Uganda National Council for Science & Technology	University of KwaZulu-Natal Biomedical Research Ethics Committee	National Health Research Review Board

a *For men who had female partners enrolled in DREAMS, eligible men were those whose female partners had voluntarily listed them and consented that DREAMS implementing partners could make contact, and who DREAMS implementing partners had already contacted to offer HIV services. Other referrals included men recruited from potential HIV transmission areas, identified by key informants as places where men and women tended to meet casual sex partners*.

b *A 2017–2018 cross-sectional survey administered by the parent study on which men self-reported their HIV status (34)*.

c *Approximate equivalence in US dollars is shown; incentives were provided in the local currency*.

Study sites within each country were selected in consultation with PEPFAR colleagues, DREAMS implementing partners, and local stakeholders and included high HIV prevalence urban and peri-urban/rural areas where DREAMS HIV-prevention programs were being implemented. These areas were intended to be geographically representative of DREAMS program communities (33). We used purposive sampling to recruit participants to support our aim of better understanding typical experiences of MLHIV across multiple country settings (35, 36). To be eligible for the study men had to be at least 18 years old, and willing and able to provide informed consent to participate. Most participants were known to be HIV-positive at the time of recruitment and were recruited in part because of that status. In a few cases, participants self-disclosed their HIV-positive status during the course of the interview. The studies were approved by the Population Council Institutional Review Board (New York, USA) and country-specific institutional review boards. Additional information about study methods employed in each country, including study sites, recruitment strategies, data collection, incentives, and ethical approval, are presented in Table 1.

Semi-structured in-depth interview (IDI) guides (focus group discussion [FGD] guide in Malawi) were similar in each country and assessed men's experiences and perspectives regarding HIV testing, care, ART use, and viral load suppression. Participants also provided basic sociodemographic information. IDIs and FGDs were conducted in private locations by trained, local interviewers in a local language or English. IDIs and FGDs lasted 30–90 min, were audio-recorded, translated into English if needed, and transcribed verbatim by research staff.

Transcribed IDIs and FGDs were imported into Atlas.ti (37) for coding and analysis. A comprehensive codebook was developed using deductive (based on a priori research objectives) and inductive (based on emergent themes from the data) methods. Three trained researchers each coded a sample of interviews that were subsequently reviewed by the rest of the team for consistency, internal validity, and resolution of disagreements. Reports for each code, stratified by country, were generated and reviewed to aid analysis, using a constant-comparative approach (38).

## Results

**Figure 1:**
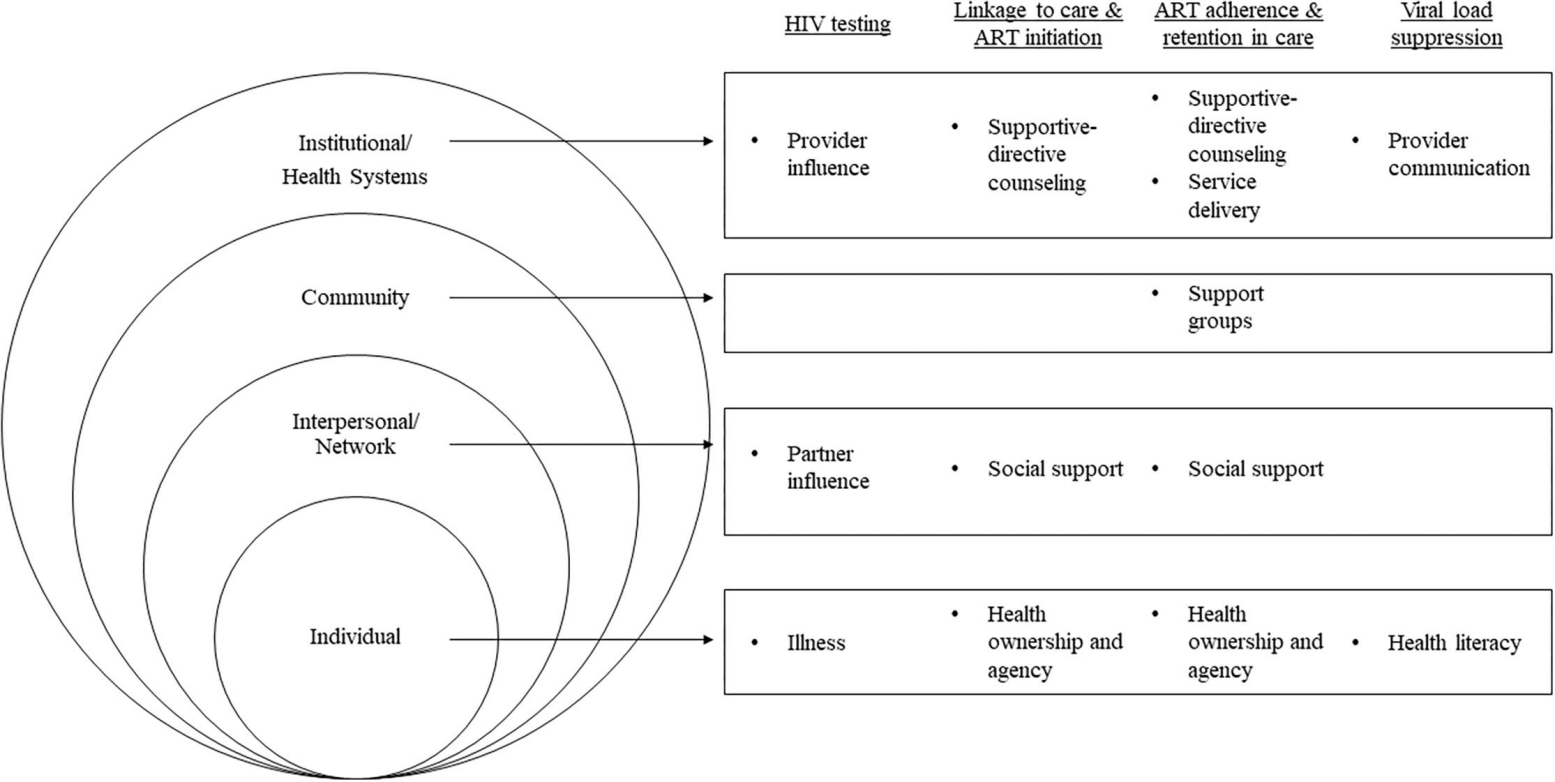
Socioecological-level facilitators supporting MLHIV across the HIV-care continuum in four sub-Saharan African countries, 2018–2019 (*N* = 92).

Socio-demographic characteristics of the 92 study participants are presented in Table 2. Participants in Eastern Africa were, on average, several years older than participants in Southern Africa, and a higher proportion reported being married. Roughly two thirds or more reported being employed in all countries except South Africa, where less than a third did. Employment in farming, sales, business, civil service, and craftsmanship were commonly reported. Themes that emerged at each stage of the HIV-care continuum are described below with representative quotes and are depicted by their respective socioecological level in Figure 1 (I: Interviewer; R: Respondent).

**Table 2:** Sociodemographic characteristics of MLHIV in four sub-Saharan African countries, 2018–2019 (*N* = 92).

	**Eastern Africa**		**Southern Africa**	
	**Malawi** **(*n =* 48)**	**Uganda** **(*n =* 17)**	**South Africa** **(*n =* 20)**	**Eswatini** **(*n =* 7)**
Median age in years (range)	39.5 (21–72)	39 (23–54)	30.5 (23–40)	31 (24–33)
Married, *n* (%)	41 (85%)	13 (76%)	5 (25%)	4 (57%)
Employed, *n* (%)	45 (94%)	11 (65%)	5 (25%)^a^	5 (71%)

a *Employment information was unavailable for roughly 30% of South African participants*.

### HIV Testing

Most men in each country reported testing positive for HIV within the past 5 years. One respondent did not believe that he was living with HIV, doubting the accuracy of his previously positive test result; another did not disclose his status but indicated he was taking ART. In Malawi, South Africa, and Eswatini, many reported that their positive HIV test was their first-ever HIV test; testing history was not fully assessed in Uganda.

#### Illness and Provider/Partner Influence

Illness was the dominant facilitator of testing in South Africa, Malawi, and Eswatini, and also emerged among a minority of men in Uganda (although only about one-third of the sample in Uganda provided information on testing motivation). Two patterns dominated: (1) illness driving men to seek out HIV testing directly, as the men may have suspected their illness was related to HIV, and (2) illness driving men to seek medical care for the illness, during which healthcare providers directed men to test for HIV. For some men, the illness that ultimately led them to get tested was serious and recurrent, incapacitating them in some instances.

*R: The reason I went for HIV testing was because I was not feeling well and losing weight…. I was very sick.... Diarrhea nearly killed me. I almost died of dehydration. I was feeling very weak. I could not stand on my own feet. I was a mess*. (South Africa, age 29, single with partner)

*R: The disease showed itself through tuberculosis…. Then immediately [healthcare workers] told me that, “We should take your blood sample for HIV.” After testing, they confirmed that I had HIV*. (Uganda, age 54, married)

For a subset of men in Malawi, illness was present but insufficient to drive them to test for HIV. Encouragement by their female partners or individuals in their social network (e.g., friends) provided additional motivation for them to test.

*R: I was frequently getting sick; however, I was afraid to come to the hospital. But my wife said, “My husband, the way you are, we have to go to the hospital.” So, we went…and then [after a positive result] I started receiving medication*. (Malawi, age 40, married).

Another subset of men in Malawi reported getting tested due to their female partner's HIV-positive diagnosis.

### Linkage to Care and ART Initiation

The majority of men across countries described immediate linkage to HIV care. This included immediate initiation on ART, or immediate initiation on Bactrium plus CD4-count monitoring through follow-up testing, depending on the local ART initiation policies at the time of diagnosis. All but 3 of the 92 men reported currently taking ART. Few men specified the increment of time between diagnosis and linkage to services; among those who did, the range was roughly 1–30 days. Two men acknowledged that they had chosen to delay treatment after initial diagnosis due to self-described denial about the result, but explained that they had since accepted their positive status and initiated ART.

#### Supportive-Directive Counseling

Across countries, men described that they decided to initiate ART due to having received counseling from healthcare providers. Men's accounts suggest providers utilized a supportive-directive counseling style consisting of three components, described alone or in combination: (1) empathic support and encouragement, especially at the time of diagnosis, (2) directive counseling, in which providers directly suggested that participants complete a specific course of action, such as initiate ART, and/or (3) educational counseling, in which providers conveyed information that enhanced participants' understanding of HIV and ART, such as the importance of early ART initiation.

*R: [At diagnosis, the counselor] told me that, now that I am HIV-positive…I can still cope with life and be a man like everyone [else]. I can still have my own family…. [The counselor said] I should take medication at one specific time every day. He also talked about the change in my lifestyle and the way I should eat; if I am smoking, drinking alcohol and sleeping around with girls, all that should stop*. (Eswatini, age 30, single)

*R: It was clinic counselors during the seven-day classes [that helped me decide to start ART]. They give you genuine information during these classes, so that by the time you leave, you are able to separate facts from the stories, as far as HIV and ARV-treatment goes*. (South Africa, age 31, single)

#### Social Support

Social support outside of the healthcare setting also proved effective in ART initiation, particularly in Malawi. Men recounted how they had received encouragement to start ART from friends, family, and/or intimate partners, and how they had followed through with initiating ART as a result. This was minimally present in the other countries.

*R: [My friend] was surprised with my body and the way I was working. He is the one who encouraged me that, “The way you used to work – that time, and this time – it*'*s different. So, rush to the hospital.” I understood when I went there it was true because I was diagnosed with the virus. I thank him; he is also in the support group. I don*'*t know – the power of God showed him to me, to encourage me to go to the hospital and get treatment on time*. (Malawi, age 43, married).

*R: [I started ART] when I realized that some of my relatives were also on ART treatment that I was not aware of. They explained to me themselves and encouraged me not to be worried that I tested positive for HIV virus. They also started treatment way back before a lot of people started in the area*. (Malawi, age 24, married)

#### Health Ownership and Agency

Men in Eswatini, Malawi, and Uganda described their decision-making about ART initiation in ways that conveyed a sense of ownership and agency regarding their health. They described how they had initiated ART at their own direction, and often verbalized ART's expected benefits to their health and longevity as particularly motivating.

*R: When I finally came to and confirmed my status…they asked me whether they could start me on treatment. I said there is no negotiation over that; it is automatic that I start right away. I told them that is what brought me here*. (Uganda, age 28, single with partner)

*R: No one helped me. I made the decision on my own…considering that I was sick, I just made the decision to start treatment*. (Malawi, age 24, married)

*R: What helped is the fact that I had heard that people who were positive and were properly taking their medication were better positioned to stay healthy. Knowing that helped accept my situation*. (Eswatini, age 33, single with partner)

### ART Adherence and Retention in Care

As noted above, nearly all participants reported being prescribed ART. Those in Uganda, South Africa, and Eswatini reported taking it as directed, noting similar facilitators as for ART initiation. A few men described past changes to their ART regimen, some due to non-adherence that produced treatment-resistance and some due to adverse side effects. In Malawi, though men described being able to take ART daily, they noted difficulties adhering to the related dietary requirements because they could not afford to purchase recommended foods.

#### Supportive-Directive Counseling

Supportive-directive counseling was influential in men's ART adherence. Participants described being counseled on the numerous lifestyle changes necessary to support ART adherence and to maximize ART's effectiveness, such as eating a healthy, well-balanced diet, drinking lots of water, exercising, wearing condoms, and reducing or stopping all substance use. However, many participants who reported having implemented lifestyle changes to support their HIV care and health did not explicitly link these changes to counseling.

*R: [During initial care visits, the provider/counselor and I] usually talked about positive living. This includes sexual behaviors, foods to eat, and how to take medication correctly. They said if I am smoking or drinking, I should quit because that will disturb my memory; when drunk I will forget taking medication. Also, they said I should avoid fatty or fried foods but eat healthy foods, not junk, and I must not take medications on an empty stomach. Most important, I should take medications on one specific time, for example if it is 7pm it has to be that time every day*. (Eswatini, age 30, single)

*R: I*'*ve not found any problem [with taking ART] yet. As long as you follow the guidelines from the health providers then you will not find any problem. They stop you from using harmful drugs like alcohol and cigarettes. I have been following the health providers*' *guidelines, and I have not found any health problem so far*. (Uganda, age 41, married)

*R: I do not have any complaints [taking ART]. My system is responding very well. I guess it*'*s because I have stopped smoking and alcohol*. (South Africa, age 29, single with partner)

At follow-up and refill visits, participants had discussions with providers on adhering to ART and were encouraged to continue adherence, being occasionally reminded to maintain “positive living” (e.g., healthy diet, etc.) to support ART's effectiveness. However, not all men indicated receiving counseling at follow-up or refill visits, and others indicated they intentionally skipped such counseling.

*R: [During my initial regular-care visits,] we would talk about my treatment – if I*'*m still taking my medication the way I*'*m supposed to. She would also encourage me to take care of myself and make sure I take my medication properly and on time and make sure I finish my medication and that finishing them is important. [At follow-up visits], they would count the tablets [to see] if I have been taking them properly. The counselor would encourage me to come back to the clinic on time and also to take my medication*. (Eswatini, age 31, single)

*R: [Counselors] advise me about positive living, what to do in order to stay healthy. They also inquire about my adherence to drugs. They talk about nutrition, the types of foods that I eat, and the work that I do when I am at home*. (Uganda, age 25, single with partner)

Many men indicated their ART adherence was simply a matter of doing “as [they were] told,” “as instructed,” or “as the doctor recommended,” particularly in cases where educational counseling was provided.

*R: At first, when the doctor was giving us the medication, they said that, “These medications – you are supposed to be taking them for the rest of your life. When you stop taking them, you will also start getting sick. So, once you start getting sick again and if you come here, we will give you the medication, but those medicines might not work. Because it might happen that the virus has become resistant and may not be affected by the medicine. So, you are placing your life in danger when you stop.” So, because of the love that I have for my life, I take them on a daily basis*. (Malawi, age 42, married)

#### Service Delivery

Across countries, several aspects of service delivery emerged as important facilitators of men's continued, positive engagement in HIV care. Men appreciated services they perceived to be responsive to their immediate medical needs, including those related to HIV and other matters. They also appreciated services they perceived to be quick and confidential, and where staff treated them with kindness and respect.

*R: What makes me contented is that, for example, first time I came, all the doctors, nurses and people here…welcomed me nicely…. They counseled me in a lovely manner; they did not hide anything from me. When it*'*s my date to get the drugs, they welcome me cheerfully*. (Malawi, age 27, married)

*R: It*'*s no longer like in the past - in the past you would stay long at the clinic, but now it is better because you come in now and leave now*. (South Africa, age 32, single)

The salience of each aspect of service delivery varied somewhat from country to country (e.g., privacy was noted as important in all countries except Uganda; quick services were noted in Southern but not Eastern African sites). These features of service delivery enhanced men's overall satisfaction with their care and in some cases influenced their choice of clinic.

#### Social Support

Participants from Malawi were members of HIV support groups, in which adherence support was a main objective of the groups. Men attending the groups held one another accountable and provided encouragement for remaining adherent to ART.

*R: [In] the support groups, we are able to encourage one another. So, when you hear encouragement like this...you tell yourself that, “I must continue taking them” [i.e., ART]*. (Malawi, 42, married)

Receiving adherence-related social support outside of the clinical/support group setting was evident among most participants from Uganda but was minimal among participants in the other countries. Among Ugandan participants, while some of the relationships that provided such support involved family members, most involved intimate partners, some of whom were also HIV positive. Examples of support included encouragement and reminders to keep HIV care appointments and to take ART, as well as assistance with transportation to appointments and with retrieving ART refills.

*R: My partner…helps me to adhere to HIV treatment and care. She reminds me of my appointment dates with the health workers, also of when I am supposed to take my medicine*. (Uganda, 46, married)

*R: When my appointment date is due, if I don*'*t have money, I call [my sister] because she stays on that side of town. So, I ask her to go help me collect the medicine...then she sends it to me*. (Uganda, 35, married)

A few participants with an HIV positive partner described how they and their partner held one another accountable to adhere to ART.

#### Health Ownership and Agency

In Malawi, South Africa, and Eswatini, health ownership emerged as an important facilitator of ART adherence. Phrases such as “I make sure” were commonly used when discussing adherence and suggest the responsibility many men felt to carefor their health.

*R: I make sure that I take my medication every day…. I am profoundly serious about taking my treatment…. I do not have time for games, and I never get confused, and I know what I am doing. Nothing can disturb me now*. (South Africa, age 31, single with partner)

*R: Nothing gets in the way of taking my medication. I go to take medication so that life goes on, and I am also stronger than before*.

*I: There*'*s nothing that makes you to forget?*

*R: No, I have never forgotten*.

*I: Is there any person that encourages you maybe to go get medication?*

*R: Me. Myself. When I get home, I take my medication. After taking medication for some time, I check in the book which they gave me that on such day I am supposed to go and receive medication*. (Malawi, age 36, married)

Men also explained how they had developed their own strategies to adhere on a day-to-day basis. Some men used cellphone alarms and television programs as reminders to take ART, while others had to go to further lengths to ensure they would not miss any dose of treatment.

*R: I follow the instructions given by the doctor, that we should always take the drugs at 7 o*'*clock after having our supper and before going to bed. I have never skipped not even for a day. Even if I am going somewhere or I am going to work, they are always with me. When I am going somewhere where I will spend a night or to my relative, I carry them in a bottle. If I am traveling, I have to ask for a transfer. I also have to make sure that I have access to the hospital to the place where I am going where I could be able to get my drugs so that I don*'*t skip*. (Malawi, age 45, married)

### Perceived Viral Load Suppression

Respondents' perceived achievement of viral load suppression was near-universal among those who reported knowing their viral load. However, this could not be accurately assessed among all participants due to limited knowledge about viral load, generally, and their own viral load testing experiences, specifically. Given these limitations with assessing viral load, we report both facilitators and barriers to awareness, understanding, and perceived achievement of viral load suppression.

#### Provider Communication and Health Literacy

Roughly 3 in 4 men in Uganda knew they had undergone viral load testing and reported being virally suppressed, with some specifically linking viral load suppression to ART adherence. In addition, almost half of Ugandan participants demonstrated a clear understanding of treatment as prevention (TasP), many of whom credited such understanding to healthcare workers.

*R: I was told that the virus in my body is no longer active, and I was told that this is a result of my being consistent with taking ARVs*. (Uganda, age 40, married)

*R: The counselors tell us, when we go at the health center...that when you consistently take your medication, you have an opportunity to live longer and that there is 90% chance that if you have been consistent in taking your medicine, when you sleep with an uninfected partner, she may not get the virus, because the medicine they give us suppresses the virus and it sleeps so that it*'*s not active to infect the other person*. (Uganda, age 27, married)

Few men in South Africa or Eswatini knew for certain whether they had received viral load testing, and those who did could remember few details (e.g., most recent viral load test, results of most recent test, whether or not they were told the results). Some participants described how providers tended not to explain the various tests being performed or divulge the results of those tests. Despite the lack of knowledge regarding one's own viral load test, when asked directly, a handful of the same participants exhibited some understanding of the meaning of viral load suppression and its implications for preventing HIV transmission to sexual partners.

*I: What do you know about viral load testing?*

*R: No one really explained thoroughly what it is*.

*I: How many times have you done viral load testing?*

*R: I think I have done it like six times*.

*I: When was the last viral load testing?*

*R: I cannot recall*. (Eswatini, age 33, single)

*I: Do you know that ART can prevent HIV from spreading to your partner? For instance, let*'*s say you are dating an HIV negative partner and you*'*re positive and you take your treatment very well. In your own knowledge, can you infect your partner?*

*R: If you do not use a condom while having sexual intercourse?*

*I: Yes*.

*R: Yes, it is possible depending on how strong they are, but I do not think it will be 100%. It can only depend on how strong my partner is*. (South Africa, age 39, married)

For those who did not demonstrate an awareness of TasP, when presented with the information, replies included “I didn't know that” and “I have never heard of such.”

In Uganda, over half of participants confirmed that they had previously had their CD4 count checked, though few recalled or reported the actual number. In South Africa and Eswatini, more participants were aware of having had their CD4 count checked than their viral load tested, but few recalled or reported the actual number. Issues related to viral load, CD4, and TasP were not assessed in Malawi.

## Discussion

We identified socioecological-level facilitators of engagement in services across the HIV-care continuum among MLHIV in Malawi, Uganda, South Africa, and Eswatini, including health ownership and agency (individual level), social support (interpersonal/network and community level), supportive-directive counseling (institutional/health systems level), and male-friendly service delivery (institutional/health systems level). While men described progress in the middle of the continuum (i.e., ART initiation and adherence), they described challenges at the start and end of the continuum (i.e., timely HIV testing and awareness/achievement of viral load suppression). Facilitators that supported men's progress in the middle of the continuum could potentially help men overcome challenges at either end, potentially improving HIV testing and viral suppression.

A notable facilitator of ART initiation and adherence at the individual level was having a sense of ownership and agency over one's health, which has previously been posited as a key factor promoting HIV testing and living well with HIV (20, 39). Some research has highlighted the potential emasculating power of both HIV (2, 40, 41) and healthcare utilization (12, 42, 43) as barriers to men's engagement with HIV services. For men in our sample, taking responsibility over their health and exercising control over HIV and their future may have been more of a reflection of their masculinity rather than a forfeiture of it (44, 45). Further promoting this sense of health ownership among our participants were the health benefits they experienced from taking ART. Consistent with other research in Uganda and Kenya (46), the men noticed and appreciated ART's effect on improving or safeguarding their health—likely quite dramatic given that persistent illness had prompted men in our sample to seek care—and wished to maintain and extend those effects. Cultivating ownership and agency over HIV-status and related wellbeing among MLHIV may be an important direction for HIV programming (e.g., applied to messaging campaigns about ART's benefits).

Consistent with other studies with MLHIV from SSA (46, 47), social support was linked to ART initiation, adherence, and care-retention among our participants, especially in Malawi and Uganda, where marital relationships were more common than in Southern African sites. These findings show how consequential men's HIV status disclosure to their partners is, as this facilitated a range of partner supports that contributed to men's successful use of services. Men specifically described the importance of non-clinical informational and instrumental social support, both of which have been shown to help individuals cope with living with HIV (48–50). This may therefore be important to target in future programming for men. Also consistent with prior research with MLHIV in SSA (51), providers played multiple roles for our participants, serving as clinical authorities, HIV experts, emotional supports, and health-status monitors. In contexts such as South Africa and Eswatini where MLHIV have limited interpersonal support, leveraging existing social networks (52), promoting the development of and providing access to new ones (53), and strengthening relationships with providers may be critical.

Other factors that facilitated participants' service-use were institutional in nature—including service delivery that was rapid, welcoming, and private—and reflect recent qualitative work with MLHIV in Kenya (20). Rapid services require little time away from work and home (19); private services can prevent or mitigate experiences of stigma (2, 41); and a welcoming environment may counter anticipated stigma and expected social repercussions for being sick and vulnerable (54). Additional research to understand specifically those aspects of HIV care and treatment service delivery the men perceived as rapid, private, and welcoming is needed. However, it is clear that male-friendly service delivery models that are responsive and adaptive to men's unique health needs and wants as patients can better facilitate their engagement in HIV services and broader HIV-prevention efforts (1, 10, 25).

Accounts of timely, consistent engagement with HIV services in the middle of the HIV-care continuum contrasted with those at the beginning, as most respondents in South Africa, Eswatini, and Malawi had tested for HIV only after becoming ill or having been persistently ill. Notably, this was within the last 5 years, when targeted efforts to reach and test men in high-prevalence regions were underway (55, 56), perhaps demonstrating how these efforts have in fact been reaching men, particularly men who have been living with HIV for some time. This pattern of testing was less evident in our findings from Uganda, where HIV testing prevalence tends to be relatively high (57, 58). Determining facilitators to high HIV-testing prevalence in Uganda and applying them to comparable SSA contexts may be useful for addressing HIV testing challenges for men.

Collectively, our findings may reflect experiences of harder-to-reach MLHIV and may account for the gaps in achieving some of the 90-90-90 indicators across the sites represented here: Malawi, where 72% of MLHIV know they are positive and 89% have initiated ART (59); Uganda, where 67% of MLHIV know they are positive and 87% having initiated ART (60), South Africa, where 78% of MLHIV know they are positive and 67% have initiated ART (61); and Eswatini, where 80% of MLHIV know they are positive and 90% have initiated ART (62). Differentiated service delivery models (63, 64), such as community-level testing, could be instrumental in reaching these men. Community-based testing options (e.g., mobile, home, self-testing) that have proved effective in prior research, especially those that have demonstrated highest yield in a given context, could be particularly instrumental (16, 18, 65–68).

Challenges were also evident at the end of the HIV-care continuum. Many respondents had no or minimal knowledge about viral load testing and viral load suppression, or their own viral load testing history and viral load status. Moreover, knowledge about the implications of viral load suppression for their own health and for reducing the likelihood of transmission to their sexual partners was lacking. Efforts to expand regular viral load testing in the region, efforts by providers to educate patients about treatment and viral load suppression, and patient demand for this information have been uneven (2, 69, 70). Expanding coverage of regular viral load testing, training providers to effectively convey viral load-related information to MLHIV, equipping HIV-support groups with informational resources on ART and viral load, and empowering men to take an equally active role in their HIV care may support viral load suppression and increase health literacy.

Facilitators identified in this study could be leveraged to address challenges at the start and end of the HIV care continuum. There were several positive, multilevel facilitators of ART initiation and adherence, while there was primarily one (negative) facilitator of HIV testing—illness, with the other HIV testing facilitators occurring only in response to illness. Similarly, facilitators of perceived viral load suppression were few with minimal impact. Psychosocial interventions informed by socio-behavioral frameworks and theory (39, 71, 72) that target men's underlying motivations to cultivate health ownership and agency may enable earlier testing, particularly among resistant testers (73). Similarly, leveraging the support of men's existing social networks (especially for men without partners), creating or strengthening community support groups, and developing service-delivery and supportive-directive counseling toolkits for providers and healthcare staff may support earlier HIV testing and viral load suppression. Application and evaluation of these strategies in high-prevalence areas in different country contexts could help close gaps in HIV testing and viral load suppression.

This study has several limitations. Recruitment, sampling methods, and sample sizes differed to some extent across sites, which may have limited comparability of findings across contexts. Similarly, some HIV-related topics were not uniformly addressed across settings in IDI/FGD guides, nor were sociodemographic characteristics (e.g., family size, income, education, tribe) that could serve to better contextualize findings. Second, the financial incentive may have influenced who elected to participate in the study. However, participant compensation was in accordance with institutional review boards of record for each site and commensurate with comparable studies in the country context. Third, given the qualitative nature of the research, including the small and disparate sample sizes across countries, findings may not be generalizable to MLHIV in other contexts. Fourth, though near-universal current ART use helped us identify facilitators of ART-initiation and adherence, it also limited our ability to contrast those on ART with those not on ART. Finally, limited awareness of viral suppression does not necessarily indicate lack of actual viral suppression. Viral suppression is likely high given near-universal ART use, though some concern is warranted given the lack of awareness and understanding described in the results.

Our findings provide a multi-country understanding of current facilitators of MLHIV's entry into and retention in HIV care, facilitators that could be leveraged to encourage early HIV diagnosis and viral suppression. Men's engagement in HIV care and treatment is imperative to ensure health and wellbeing among themselves and their partners, and to meet global goals of ending the epidemic by 2030. Promising strategies include promoting men's sense of health ownership; restructuring health systems to ensure provision of quick, confidential, affirming services; encouraging and equipping providers to offer supportive-directive counseling, as well as education on TasP, viral suppression, and other treatment literacy concepts; and tapping key influencers in men's intimate relationships, social circles, and communities for support. Being responsive to the unique health needs of men can facilitate greater progress toward global goals of ending the HIV epidemic by 2030.

## Data Availability Statement

The datasets presented in this article are not readily available because datasets contain identifying information. De-identified transcripts will be published and tagged in Dataverse. Access will be granted after a reasonable request and statement of intended use is provided. Requests to access the datasets should be directed to dataverse.org.

## Ethics Statement

The studies involving human participants were reviewed and approved by Population Council Institutional Review Board (New York, USA) and country-specific institutional review boards. The patients/participants provided their written informed consent to participate in this study.

## Author Contributions

JW: conceptualization, formal analysis, visualization, writing—original draft preparation, and writing—review and editing. SM: study design and conceptualization, supervision, writing—review and editing, and project management. AG and NP: conceptualization, study design, tool development, data collection management, codebook development, and writing—review and editing. JP: conceptualization and writing—review and editing. All authors contributed to the article and approved the submitted version.

## Funding

This work was funded by the Bill & Melinda Gates Foundation. Funding for the Malawi study was provided by the generous support of the American people through the United States President's Emergency Plan for AIDS Relief (PEPFAR) and the United States Agency for International Development (USAID) under Project SOAR (Cooperative Agreement AID–OAA–A−14–00060).

## Conflict of Interest

The authors declare that the research was conducted in the absence of any commercial or financial relationships that could be construed as a potential conflict of interest.

## Publisher's Note

All claims expressed in this article are solely those of the authors and do not necessarily represent those of their affiliated organizations, or those of the publisher, the editors and the reviewers. Any product that may be evaluated in this article, or claim that may be made by its manufacturer, is not guaranteed or endorsed by the publisher.
